# Investigation of multiphasic 3D‐bioplotted scaffolds for site‐specific chondrogenic and osteogenic differentiation of human adipose‐derived stem cells for osteochondral tissue engineering applications

**DOI:** 10.1002/jbm.b.34542

**Published:** 2019-12-27

**Authors:** Liliana F. Mellor, Rachel C. Nordberg, Pedro Huebner, Mahsa Mohiti‐Asli, Michael A. Taylor, William Efird, Julia T. Oxford, Jeffrey T. Spang, Rohan A. Shirwaiker, Elizabeth G. Loboa

**Affiliations:** ^1^ Joint Department of Biomedical Engineering University of North Carolina at Chapel Hill and North Carolina State University Raleigh North Carolina; ^2^ Department of Biomedical, Biological and Chemical Engineering, College of Engineering University of Missouri Columbia Missouri; ^3^ Edward P. Fitts Department of Industrial and Systems Engineering North Carolina State University Raleigh North Carolina; ^4^ Department of Orthopaedics University of North Carolina School of Medicine Chapel Hill North Carolina; ^5^ Biomolecular Research Center Boise State University Boise Idaho

**Keywords:** 3D‐printing, chondrogenic differentiation, human adipose derived stem cells, osteochondral, osteogenic differentiation

## Abstract

Osteoarthritis is a degenerative joint disease that limits mobility of the affected joint due to the degradation of articular cartilage and subchondral bone. The limited regenerative capacity of cartilage presents significant challenges when attempting to repair or reverse the effects of cartilage degradation. Tissue engineered medical products are a promising alternative to treat osteochondral degeneration due to their potential to integrate into the patient's existing tissue. The goal of this study was to create a scaffold that would induce site‐specific osteogenic and chondrogenic differentiation of human adipose‐derived stem cells (hASC) to generate a full osteochondral implant. Scaffolds were fabricated using 3D‐bioplotting of biodegradable polycraprolactone (PCL) with either β‐tricalcium phosphate (TCP) or decellularized bovine cartilage extracellular matrix (dECM) to drive site‐specific hASC osteogenesis and chondrogenesis, respectively. PCL‐dECM scaffolds demonstrated elevated matrix deposition and organization in scaffolds seeded with hASC as well as a reduction in collagen I gene expression. 3D‐bioplotted PCL scaffolds with 20% TCP demonstrated elevated calcium deposition, endogenous alkaline phosphatase activity, and osteopontin gene expression. Osteochondral scaffolds comprised of hASC‐seeded 3D‐bioplotted PCL‐TCP, electrospun PCL, and 3D‐bioplotted PCL‐dECM phases were evaluated and demonstrated site‐specific osteochondral tissue characteristics. This technique holds great promise as cartilage morbidity is minimized since autologous cartilage harvest is not required, tissue rejection is minimized via use of an abundant and accessible source of autologous stem cells, and biofabrication techniques allow for a precise, customizable methodology to rapidly produce the scaffold.

## 1 INTRODUCTION

Osteoarthritis (OA) is a degenerative joint disease that limits mobility of the affected joint due to the degradation of articular cartilage and changes in the adjacent subchondral bone. OA has been estimated to affect approximately 27 million people in the United States, and the prevalence is only expected to grow (Kotlarz, Gunnarsson, Fang, & Rizzo, 2009). There are significant challenges with treating cartilage and osteochondral degradation due to the limited regenerative capacity of articular cartilage, lack of understanding of the molecular mechanisms that trigger early changes in cartilage homeostasis, and lack of tools to detect the disease at early stages. To date, there is no cure for OA and therapeutic treatments are limited to controlling pain and inflammation of the joint.

Current clinical treatment options for cartilage repair include autologous chondrocyte implantation (ACI), microfracture, and mosaicplasty (Revell & Athanasiou, 2009). ACI requires a surgical biopsy of the patient's healthy articular cartilage, followed by isolation and expansion of chondrocytes for several weeks in culture. A second surgery reintroduces the expanded chondrocytes into the defect site. However, this method has associated donor site morbidity and yields fibrocartilaginous tissue which is prone to degeneration (Niemeyer et al., 2008). In microfracture, small holes are drilled into the defect to allow mesenchymal stem cells to migrate from the subchondral bone yielding new tissue formation (Yousefi, Hoque, Prasad, & Uth, 2015). This technique also results in fibrocartilage formation (Gilbert, 1998), and therapeutic effects are only sustained for approximately 1 year (Yousefi et al., 2015). Mosaicplasty transfers plugs of autologous osteochondral tissue from nonweight‐bearing healthy regions to repair the defect. However, this method is limited by the amount of donor sites and is also associated with donor site morbidity (Getgood, Brooks, Fortier, & Rushton, 2009). Because of the limitations with current treatment options for osteochondral injury, tissue engineering approaches have become an active area of research.

One of the critical challenges from a clinical application perspective is creating an engineered tissue that can mimic the complex multiphasic nature of osteochondral tissue (Atesok et al., 2014). Because of these challenges, many stem cell‐based approaches for cartilage repair have focused on cartilage regeneration (Getgood et al., 2009; Hillel et al., 2010; Huang, Farrell, Kim, & Mauck, 2010; Huang, Farrell, & Mauck, 2010; Huang, Reuben, D'Ippolito, Schiller, & Cheung, 2004; Huang, Stein, & Mauck, 2010; Lee et al., 2008; Mobasheri, Csaki, Clutterbuck, Rahmanzadeh, & Shakibaei, 2009; Valonen et al., 2010; van Osch et al., 2009) instead of full osteochondral tissue regeneration (Deans & Elisseeff, 2009; Grayson, Bhumiratana, Grace Chao, Hung, & Vunjak‐Novakovic, 2010; Sundelacruz & Kaplan, 2009; Swieszkowski, Tuan, Kurzydlowski, & Hutmacher, 2007). Biphasic osteochondral scaffolds have been generated by assembling two separate scaffolds of engineered bone and cartilage tissue (Hung et al., 2003; Lima et al., 2004; Schaefer et al., 2000). A disadvantage of this method is the lack of mechanical integration at the junction, leading to delamination of the layers. One approach to avoid delamination is to develop a monolithic scaffold with two phases that can be cultured as a unit to induce osteogenesis and chondrogenesis simultaneously. In this method, there are challenges to promoting both chondrogenic and osteogenic factors within the same culture system (Shimomura, Moriguchi, Murawski, Yoshikawa, & Nakamura, 2014), which can be addressed by introducing biomimetic factors to the scaffold that induce either cartilage or bone formation (Mellor et al., 2015; Tampieri et al., 2008). In addition to biphasic systems, a few studies have attempted to achieve a graded transition zone of bone to cartilage throughout the depth of an engineered osteochondral tissue (Erisken, Kalyon, & Wang, 2010; Jiang et al., 2010; Kon, Mutini, et al., 2010; Kon, Delcogliano, Filardo, Fini, et al., 2010; Kon, Delcogliano, Filardo, Pressato, et al., 2010; Tampieri et al., 2008) as an attempt to improve load‐bearing capabilities in vivo and reduce shear stresses at the biomaterial interfaces (Lu, Subramony, Boushell, & Zhang, 2010). Some of the technical and culturing challenges include identifying the ideal cell source, optimizing growth factors, and signaling molecules to induce site‐specific differentiation, inhibiting hypertrophy, and developing a simple and reliable manner to manufacture scaffolds of varying size to fit a specific injury site. After implantation, the primary concern is blood vessel invasion into the cartilage region that results in endochondral ossification, and proper integration with the host tissue (Nukavarapu & Dorcemus, 2013).

**Figure 1:**
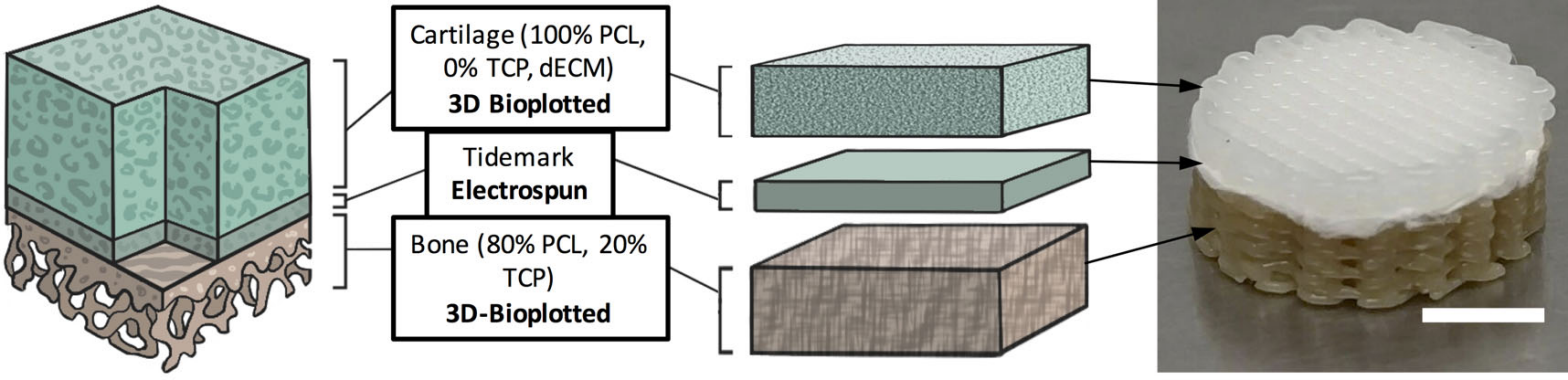
Scaffold design for osteochondral tissue generation using a single cell source. In order to engineer a full osteochondral tissue using a single cell source, site‐specific chemical cues were incorporated within the scaffold to induce site‐specific differentiation of hASC. To achieve chondrogenesis, hASC were seeded within a 3D bioplotted PCL scaffold containing dECM. An electrospun layer of PCL was created and used to recreate the tidemark. To achieve osteogenesis, hASC were seeded within a 3D bioplotted scaffold comprised of 80% PCL and 20% TCP. Full composite scaffold shown on top right (scale bar = 5 mm). dECM, decellularized bovine cartilage extracellular matrix; hASC, human adipose‐derived stem cells; PCL, polycraprolactone; TCP, β‐tricalcium phosphate

The objective of this study was to investigate the use of a single cell source, human adipose‐derived stem cells (hASC; Nordberg & Loboa, 2015; Pittenger et al., 1999), within an integrated multimaterial, multiscale triphasic 3D scaffold created using a combination of 3D‐bioplotting and electrospinning to achieve complete osteochondral tissue generation. Human ASC, which are more abundant and accessible than human bone marrow‐derived mesenchymal stem cells (hMSC; Awad, Wickham, Leddy, Gimble, & Guilak, 2004; Huang, Zuk, et al., 2004; Zuk et al., 2001), are capable of both osteogenic and chondrogenic differentiation and can express bone and cartilage extracellular matrix (ECM) constituents (Zuk et al., 2001). Although some reports indicate that hASC chondrogenesis may be inferior to hMSC chondrogenesis (Huang et al., 2005; Mehlhorn et al., 2006), this can be overcome by alternative growth factor supplementation (Puetzer, Petitte, & Loboa, 2010). Our triphasic 3D scaffold consists of physiologically inspired chemical cues to induce site‐specific hASC differentiation in a manner that recapitulates the depth‐dependent properties of native osteochondral tissue. The integrated 3D scaffold includes a 3D‐bioplotted deep layer of a composite of polycaprolactone (PCL) and β‐tricalcium phosphate (TCP) to induce osteogenesis, a 3D‐bioplotted superficial layer of PCL combined with decellularized articular cartilage ECM (dECM) hydrogel to induce chondrogenesis, and an intermediate electrospun layer of PCL to mimic the native tidemark of osteochondral tissue to separate the two phases and potentially prevent blood vessel invasion into the cartilage layer in vivo (Figure 1).

## 2 MATERIALS AND METHODS

We first conducted analyses of hASC osteogenesis and chondrogenesis on separate phases of 3D‐bioplotted PCL‐TCP and PCL‐dECM scaffolds, respectively, and then assessed the site‐specific hASC differentiation characteristics of integrated multimaterial triphasic scaffolds resembling full thickness osteochondral tissue.

### 2.1 Preparation of PCL‐TCP composite

The PCL‐TCP composite (80–20% by weight) used for 3D‐bioplotting of the osteogenic scaffold phase was created by mixing the appropriate proportion of PCL (*M*_n_: 80,000, ~3 mm pellets; Sigma‐Aldrich, St. Louis, MO) and TCP (Riedel‐de‐Haën AG, Seelze, Germany), and heating the mixture at 180°C for 60 min with intermittent stirring in a temperature‐controlled oven until a homogeneous, single phase mix was obtained.

### 2.2 Preparation of decellularized ECM hydrogel

Decellularized ECM derived from bovine hooves (Micro Summit Processors, Micro, NC) was used in a hydrogel form as a constituent of the 3D‐bioplotted scaffolds for the chondrogenic phase. Cartilage was harvested from fresh hooves and decellularized by suspending in a solution of deionized water and 0.5% penicillin/streptomycin, and mixing for 24 hr. The decellularized cartilage was then frozen in 1X phosphate buffered saline (PBS) and lyophilized for 24 hr. Finally, the lyophilized cartilage was pulverized in a mill using a number 40 size mesh to obtain the decellularized cartilage ECM powder.

ECM gels were prepared at an initial concentration of 25 mg/ml. The dECM was digested in a 2 mg/ml solution of pepsin in 0.1M HCl. Four milliliter of this solution was added to 100 mg of cartilage dECM powder. The ECM was digested in the pepsin solution for 48 hr. Next, 400 μl of 1M NaOH was added to the solution in order to bring the pH to 7.4. Finally, 489 μl of 10X PBS was added to the solution to make the concentrations of NaOH, HCl, and PBS equal to each other. The final concentration of the gel was calculated to be 20.5 mg/ml.

### 2.3 Proteomic analysis of decellularized ECM

Proteins were extracted using RIPA buffer protocol (Millipore, Billerica, MA). Protein concentrations were determined using a BCA protein assay (Thermo Fisher Scientific, Waltham, MA). A volume of 20 μg of total protein from each sample was digested in solution with Trypsin/Lys C mix (Promega) following the manufacturer's instructions. Briefly, protein samples were precipitated with cold acetone and dissolved in 8M urea solution. After reduction with dithiothreitol and alkylation with iodoacetamide, samples were diluted with 50 mM Tris–HCl pH 8.0, to reduce urea concentration to 1M. Trypsin/Lys C Mix was added at a 20:1 protein: protease ratio (wt/wt). Samples were digested overnight at 37°C. Digestion was terminated with trifluoroacetic acid. Resulting peptide mixtures were cleaned using a C18 reverse‐phase spin column (Thermo Fisher Scientific), dried under vacuum, and reconstituted in 5% acetonitrile, 0.1% formic acid in water for LC–MS/MS analysis.

LC–MS/MS analysis of peptides was conducted on a Velos Pro Dual‐Pressure Linear Ion Trap mass spectrometer equipped with a nano electrospray ionization source and coupled with an Easy‐nLC II nano LC system (Thermo Fisher Scientific). A volume of 5 μl of peptide mixture was loaded onto a C18 reverse‐phase column (10 cm x 75 μm, 3 μm, 120 Å). Each sample was analyzed three times. A linear gradient with two mobile phases (A: 5% acetonitrile, 0.1% formic acid, 94.9% water, 0; B: 80% acetonitrile, 0.1% formic acid, 19.9% water) at a flow rate of 300 nl/min was used to separate peptide mixtures. The gradient began at 0% B, increased linearly to 50% B over 180 min and then to 100% B over 16 min, and maintained at this percentage for 14 min as a washing step. Eluted peptides were ionized in a nano ESI source with a spray voltage of 2.2 kV. Full scan MS spectra were acquired from m/z 300 to 2000. Collision‐induced dissociation was used to fragment the precursor ions. MS/MS spectra were acquired in the data‐dependent acquisition mode for the 10 most abundant precursor ions in the preceding full MS scan.

Peptide spectral matching and protein identification were achieved by database search using Sequest HT algorithms in Proteome Discoverer 1.4 (Thermo Fisher Scientific). Raw spectrum data were used to search against the UniProtKB/Swiss‐Prot protein database for bovine. Main search parameters included: trypsin, maximum missed cleavage site of two, precursor mass tolerance of 1.5 Da, fragment mass tolerance of 0.8 Da, and variable modification of oxidation/hydroxylation of methionine, proline, and lysine (+15.995 Da). Decoy database search was performed to calculate false discovery rate (FDR). Proteins containing one or more peptides with FDR ≤ 0.05 were considered positively identified and reported. Total number of peptide spectral matches for each protein reported by Protein Discoverer 1.4 was used for quantification.

### 2.4 3D‐bioplotting of osteogenic and chondrogenic scaffold phases

We have previously optimized the geometry and assessed the biocompatibility of the 3D‐bioplotted scaffolds used in this study (Mehendale, Mellor, Taylor, Loboa, & Shirwaiker, 2017; Mellor et al., 2017). Using the methods described previously, the base computer assisted design models of the scaffolds were created in Solidworks (Dassault Systèmes SOLIDWORKS Corp., Waltham, MA) as discs (Ø14.5 x 2 mm) to fit the individual wells of a standard 24‐well culture plate. The STL file, sliced into six layers, was positioned onto the bioplotting stage (BioplotterRP, EnvisionTEC GmbH, Gladbeck, Germany). To begin scaffold fabrication on the 3D‐Bioplotter (EnvisionTEC GmbH), the strand‐pore geometry previously determined to be favorable for hASC (Mehendale et al., 2017) and experimentally optimized process parameters were assigned (Table 1) in VisualMachines. A total of 6.8 g of material (80% PCL‐20% TCP composite for osteogenic scaffolds; 100% PCL for chondrogenic scaffolds) was preheated in the high temperature cartridge and the scaffolds 3D‐bioplotted, one at a time. Postfabrication, all scaffolds were weighed (AR2140 digital balance, Ohaus Corp., Parsippany, NJ) and characterized for strand width and interstrand spacing (×50 magnification, KH‐7700 microscope, Hirox, Hackensack, NJ). From each scaffold, for each dimension metric, measurements were taken at eight random locations. All scaffolds were sterilized for 30 min in 70% ethanol, and washed three times with sterile ×1 PBS and once with complete growth medium (CGM, defined below).

**Table 1:** Scaffold design and 3D‐bioplotting parameters

		PCL‐TCP	PCL
Scaffold design	Strand lay orientation (°, layer 1/2/3)	0/120/240	0/120/240
	Strand width (μm)	378 ± 40	351 ± 33
	Interstrand spacing (μm)	602 ± 41	632 ± 55
	Weight (mg)	154 ± 24	118 ± 16
3D‐bioplotting	Extrusion temperature (°C)	130	160
	Preheat interval (min)	25	45
	Nozzle diameter (mm)	0.4	0.4
	Extrusion pressure (N/mm^2^)	0.5	0.5
	Nozzle speed (mm/s)	0.7	0.4
	Preflow delay (s)	1	1
	Postflow delay (s)	0	1
	Wait time between layers (s)	0	3

Abbreviations: PCL, polycraprolactone; TCP, β‐tricalcium phosphate.

To prepare the PCL‐dECM scaffolds for chondrogenic analyses, 200 μl of the decellularized bovine cartilage ECM hydrogel (protocol described above) was added onto each sterilized 3D‐bioplotted 100% PCL scaffold. The hydrogel‐infused scaffolds were placed in an incubator (37°C, 5% CO_2_) overnight to allow the gel to solidify, and were then placed in 24‐well plates prior to seeding with hASC.

### 2.5 Characterization of calcium release from PCL‐TCP scaffolds

PCL‐TCP scaffolds were placed into separate wells in a 24‐well plate (*n* = 3 per time point) and treated for 30 min with 70% ethanol to mimic the sterilization process used in later cell culture experiments. Ethanol treatment also increased the hydrophilicity of the scaffold for the Ca^2+^ release experiments. The scaffolds were then fully submerged in 0.5 ml of PBS and maintained in an incubator (37°C, 5% CO_2_) for the duration of the study. PBS was collected at time points of 1, 2, 7, 14, and 28 days and stored at −25°C until assayed. A Calcium LiquiColor® Assay (StanBio, Boerne, TX) was used to quantify calcium content of each sample. Using the measured weight of the scaffolds along with the molar mass of calcium ions (Ca^2+^ 40.08 g•mol^−1^), orthophosphates (PO_4_^3−^94.97 g•mol^−1^), and TCP [Ca_3_(PO_4_)_2_ 310.18 g•mol^−1^] the weight of Ca^2+^ doped within each scaffold was calculated to be 11.9 μg.

### 2.6 Human ASC isolation, expansion, and in vitro culture of scaffolds

Excess adipose tissue was collected from five female premenopausal donors (ages 24–36) in accordance with an approved IRB protocol at UNC Chapel Hill (IRB 04–1622), and hASC were isolated from the tissue as previously described by our lab and others (Bernacki, Wall, & Loboa, 2008; Bodle et al., 2014). Cells were expanded in CGM comprised of alpha‐modified minimal essential medium (α‐MEM with l‐glutamine; Invitrogen, Carlsbad, CA), 10% fetal bovine serum (FBS; Premium Select, Atlanta Biologicals, Lawrenceville, GA), 200 mM l‐glutamine, and 100 IU penicillin/100 μg/ml streptomycin (Mediatech, Herndon, VA). Human ASC were cultured (37°C, 5% CO_2_) until reaching 80% confluency changing the media every 3 days, and then passaged using trypsin–EDTA (Invitrogen). A superlot was generated by pooling equal numbers of cells from the five individual donor cell lines into a single culture vessel as previously described by our lab, and characterized for multilineage differentiation potential by culturing for 14 days in osteogenic differentiation medium (Minimum Essential Medium, alpha modified supplemented with 10% FBS, 2 mM l‐glutamine, 100 U/ml penicillin, 100 mg/ml streptomycin, 50 μM ascorbic acid, 0.1 μM dexamethasone, and 10 mM β‐glycerolphosphate) and adipogenic differentiation medium (Minimum Essential Medium, alpha modified supplemented with 10% FBS, 2 mM l‐glutamine, 100 U/ml penicillin, 100 mg/ml streptomycin,1 μM dexamethasone, 5 μg/ml insulin, 100 μM indomethacin, and 500 μM isobutylmethylxanthine), ensuring the superlot differentiation was representative of an average of the five cell lines (Bodle et al., 2014). All experiments were run using superlot passage 6 or lower.

To assess osteogenesis, PCL‐TCP scaffolds (*n* = 8) were seeded with hASC at a density of 250,000 cells per scaffold and incubated for 28 days in CGM. To assess chondrogenesis, 250,000 hASC in 1 ml of CGM were seeded onto each PCL‐dECM scaffold (*n* = 9) and cultured for 28 days in CGM (Mellor et al., 2015; Puetzer et al., 2010).

### 2.7 Osteogenic differentiation assays

Osteogenic differentiation was assessed via calcium staining and endogeonous alkaline phosphatase activity (ALP). Scaffolds were fixed for 30 min in formalin at day 28 and stained via Alizarin Red (Sigma‐Aldrich) for calcium accretion. Endogenous ALP activity was assessed at days 14 and 28. Scaffolds were collected in 1 ml RIPA buffer (Fisher). ALP was quantified with the Alkaline Phosphatase Liquicolor Test (Stanbio, Boerne, TX), using the P‐nitrophenylphosphate methodology, as described previously (Bodle et al., 2013). ALP activity was normalized to total protein content quantified using a Pierce Micro BCA Protein Assay Kit (Thermo Fisher Scientific). Additionally, an Alamar Blue assay (AbD Serotec, Raleigh, NC) was used to assess metabolic activity of the seeded cells at days 14 and 28.

### 2.8 Chondrogenic differentiation assays

#### 2.8.1 Safranin‐O staining

Safranin‐O staining was used to determine proteoglycan content of the seeded scaffolds. Scaffolds were fixed in 10% formalin and first stained with Weigert's Iron Hematoxylin stain (Sigma‐Aldrich) for 10 min, then rinsed under running tap water. They were then stained with fast green for 5 min and washed in 1% acetic acid, followed by an 8 min Safranin‐O stain.

#### 2.8.2 Alcian blue staining

Alcian Blue staining was used to determine sulfated glycosaminoglycan (sGAG) content of the seeded scaffolds. After fixation as described above, scaffolds were stained in an Alcian blue solution (Sigma‐Aldrich) for 30 min, washed under running tap water for 2 min, and rinsed with deionized water. Scaffolds were then stained with 0.1% Nuclear Fast Red (Sigma‐Aldrich) for 5 min, tap water for 1 min.

#### 2.8.3 Quantitative RT‐PCR

Quantitative real‐time RT‐PCR was used to determine relative mRNA expression changes of chondrogenic‐specific genes in seeded scaffolds with dECM or without dECM and osteogenic‐specific genes seeded scaffolds with TCP or without TCP (*n* = 5 per condition). Total RNA was extracted directly from the scaffolds after 28 days in culture. Following the TRIZOL protocol (Thermo Fisher Scientific). Briefly, the scaffolds were submerged in 1 ml of TRIZOL followed by a chloroform (Ricca Chemical Co. Arlington, TX) extraction. The precipitated solution was incubated with an equal volume of isopropanol (Sigma‐Aldrich) and 1 μl glycoblue (Thermo Fisher Scientific) at room temperature for 10 min. The solution was centrifuged, and the precipitate was washed once with 75% ethanol (Koptec) and centrifuged again to collect the RNA containing pellet. The RNA was resuspended in DEPC treated water (Mediatech) and the yield was calculated using a NanoDrop 2000 Spectrophotometer (Sigma‐Aldrich). To quantify mRNA expression, 120 ng of total RNA was reverse transcribed into cDNA using a high capacity first strand cDNA synthesis kit (OriGene, Rockville, MD) as per manufacturer's instructions. qRT‐PCR was performed using 1 μl cDNA, 10 μl SYBR Green (Applied Biosystems, Foster City, CA), 7 μl DEPC treated water (Mediatech), and 1 μl forward and reverse primer (IDT DNA) for the following genes: b‐actin 5'‐CACTCTTCCAGCCTTCCTTC‐3', 3'‐TGTAGGCGTTTCTGGACATG‐5', Osteopontin 5′‐CTCAGGCCAGTTGCAGCC‐3′, 3′‐CTCTTAACGTCACTAAACGAAAAC‐5′ ALP 5′‐GACCCTTGACCCCCACAAT‐3′, 3′‐TCCCCTGTACGTCATGCTCG‐5′ Collagen1a2 5′‐AGAGTGGAGCAGTGGTTACTA‐3′, 3′‐GAGATGACCGCTTTGGACATAG‐5′, Aggrecan 5′‐CAGGCAGATCACTTGAGGTTAG‐3′, 3′‐ACATTAGGGTCGATGATCCCTC‐5′, Sox9 5′‐TGACCTATCCAAGCGCATTAC‐3′, 3′‐ATTTGGGAGAAGTCTCGTTCG‐5′.

qRT‐PCR was performed using an ABI 7000 Sequence Detection system (Applied Biosystems). Samples were assayed in triplicate in one run (40 cycles) per gene. qRT‐PCR data were analyzed using the 2^−ΔΔCT^ method as described previously (Livak & Schmittgen, 2001) with β‐actin as the housekeeping control. Relative quantification values are presented as fold changes in gene expression relative to the control group.

### 2.9 Fabrication and in vitro hASC culture of integrated full thickness osteochondral scaffolds

#### 2.9.1 Electrospun layer fabrication

Electrospun PCL matrices, designed to mimic the function of the tidemark layer, were separately fabricated prior to 3D‐bioplotting of the full thickness multiphasic scaffold. PCL was dissolved in chloroform and dimethylformamide (3:1 ratio by volume) to create a 12% solution. The solution was mixed continuously at 80°C for 4 hr. The PCL solution was electrospun immediately after preparation at a feed rate of 0.7 μl/hr using 15 kV and then cut into discs (Ø14.5 mm × 30 μm) to match the 3D‐bioplotted scaffolds.

#### 2.9.2 Full thickness scaffold assembly

The integrated triphasic scaffolds (Ø14.5 x 6 mm) were designed to resemble the overall organization of the osteochondral complex, and the manufacturing method used has been described previously (Mehendale et al., 2017; Mellor et al., 2017). The material composition, strand‐pore geometries, and process parameters for the bone (PCL‐TCP), cartilage (PCL‐dECM), and tidemark (electrospun PCL) phases were as described above. To create the scaffolds in a single build, the PCL‐TCP phase (Ø14.5 x 4 mm, 12 layers) was bioplotted first. Immediately upon completion of the topmost PCL‐TCP layer, the process was briefly halted, and the prefabricated electrospun PCL disc (Ø14.5 mm × 30 μm) was overlaid on top of the partial scaffold. 3D‐bioplotting was then continued with the appropriate height offset adjustment to create the PCL phase (Ø14.5 x 2 mm, six layers).

A 2‐day hASC seeding protocol was used to seed the full thickness scaffolds (*n* = 3). On the first day, the scaffolds were sterilized with successive 70% ethanol and PBS washes. The chondrogenic phase was prepared by submersing the 3D‐bioplotted PCL in neutralized dECM solution and incubating at 37°C for 1 hr. After the hydrogel had solidified, the scaffold was flipped chondrogenic PCL phase up, seeded with 250,000 hASC, and incubated overnight (37°C, 5% CO_2_). On the second day, the scaffolds were flipped PCL‐TCP phase up, and seeded with an additional 500,000 hASC. The total number of cells used in these scaffolds was tripled in the full osteochondral scaffolds because they were three times thicker than the individual chondrogenic and osteogenic scaffolds. Each osteochondral scaffold was incubated in 3 ml CGM for 28 days. Media was changed and scaffolds were flipped every 2 days.

### 2.10 Characterization of full thickness osteochondral scaffolds

After 28 days of culture, full thickness scaffolds (*n* = 3 unseeded, *n* = 3 seeded) were fixed for 30 min in formalin. Total scaffold fixation and staining has been utilized previously to demonstrate calcium distribution in bone scaffolds (Lu et al., 2017; Ma et al., 2014). Scaffolds were sectioned with a scalpel into 1 mm thick cross‐sections for histological analyses. Sections were stained via Alizarin Red, Alcian Blue, and Safranin‐O/fast green, as described above.

### 2.11 Characterization of the effects of electrospun PCL tidemark matrix

PCL scaffolds were fabricated with (*n* = 4) or without (*n* = 4) an electrospun PCL tidemark matrix to evaluate cell migration characteristics. Scaffolds had an overall thickness of 6 mm, and tidemarks were added to split the scaffolds into 2 and 4 mm sections. Each scaffold was seeded by suspending 250,000 cells in 150 μl of media in a well of a 24‐well plate for 4 hr with the 2 mm scaffold section facing downwards into the cell suspension. After 4 hr, the 2 mm section was flipped upwards in a new 24‐well plate and 1 ml of media was added to the scaffold. This yielded a scaffold with the top 2 mm section seeded with cells but the bottom 4 mm section remaining unseeded. Sterile, nontissue culture treated plates were used to prevent cells from attaching to the plate. Half media changes were carried out every other day for 28 days. After 28 days, scaffolds were fixed and stained via hematoxylin and eosin (H&E) for cell nuclei and protein distribution. The scaffolds were submerged in hematoxylin for 10 min, washed under running tap water for 5 min, rinsed in deionized water, stained via Eosin for 2 min, rinsed in deionized water and immediately imaged on an EZ4D histology microscope (Leica, Wetzlar, Germany).

### 2.12 Statistical analyses

Statistical analyses were performed using Prism (version 5.0a, GraphPad Software). Student *t* test was used to assess significant differences between two groups, and ANOVA with Tukey post hoc test was used to asses significant differences among three or more groups. All Graphs are shown in the form of mean ± *SEM*.

## 3 RESULTS

### 3.1 PCL–TCP scaffolds‐induced osteogenic differentiation of hASC

**Figure 2:**
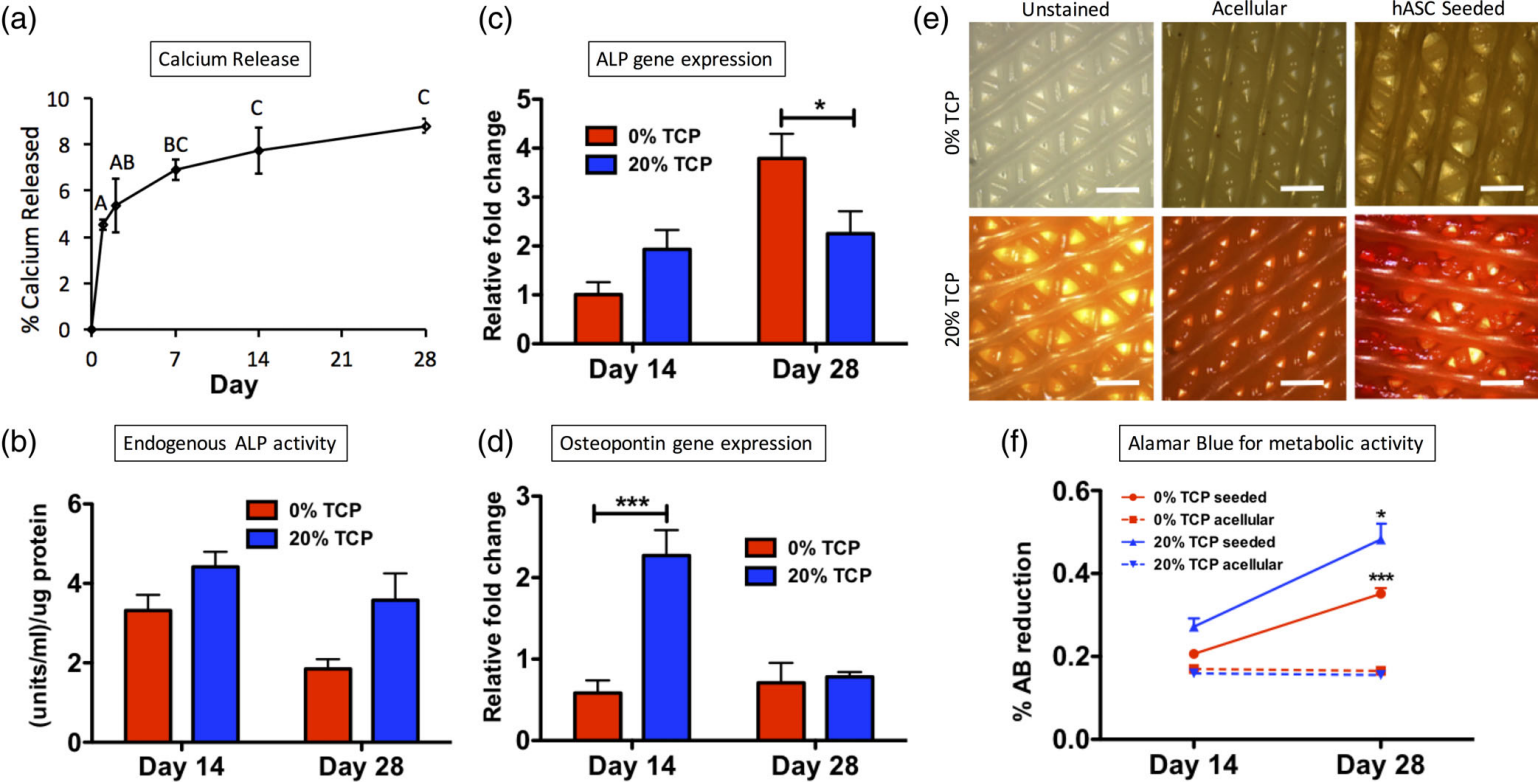
Osteogenesis of hASC on 3D‐bioplotted PCL‐TCP scaffolds. (a) The calcium release profile of osteogenic scaffolds (20% TCP 80% PCL) evaluated over 28 days (bars represent standard error of the mean; different letters indicate statistical difference [*p* < .05]). (b) Endogenous ALP activity of seeded scaffolds at both the 14 and 28 day time points. Gene expression of (c) ALP and (d) Osteopontin. (e) Alizarin Red staining indicated calcium accretion (scale bars = 200 μm). (f) Alamar Blue data indicating metabolic activity at days 14 and 28 of culture (***p < .001, *p < .05). ALP, alkaline phosphatase; hASC, human adipose‐derived stem cells; PCL, polycraprolactone; TCP, β‐tricalcium phosphate

Controlled release assays of calcium demonstrated that calcium was released from the PCL‐TCP composite scaffolds throughout the 28 day incubation period, with a large initial release within the first 24 hr (Figure 2a).

To determine how the calcium released from the scaffolds affected the seeded hASC, assays were performed to assess osteogenic differentiation. Enzymatic ALP activity, a hallmark of osteogenic differentiation (Bodle et al., 2013), was elevated PCL‐TCP composition scaffolds when compared to pure PCL scaffolds (Figure 2b). This trend was observed at both the 14 (*p* = .115) and 28‐day (*p* = .073) time points. At the gene expression level, ALP mRNA expression was elevated in PCL‐TCP scaffolds at day 14 (*p* = .323), but was reduced relative to the control by day 28 (*p = .018; Figure 2c). Osteopontin was elevated in PCL‐TCP at day 14 (*p < .001; Figure 2d). End product calcium staining demonstrated that hASC cultured on PCL‐TCP scaffolds accreted calcium while hASC cultured on PCL scaffolds alone did not accrete calcium. Acellular PCL‐TCP controls showed mild Alizarin Red staining due to the TCP found within the scaffold, but seeded PCL‐TCP stained dark red throughout the scaffold, indicating hASC‐mediated calcium accretion (Figure 2e). Scaffolds without TCP showed no Alizarin Red staining.

We have previously demonstrated that bioplotted PCL scaffolds support viability and proliferation of hASC (Mehendale et al., 2017; Mellor et al., 2017). To assess the cell viability and proliferation within the current study, Alamar Blue was used to detect metabolic activity of the seeded cells (Figure 2f). In this assay, a higher percent reduction corresponds to higher metabolic activity of the assayed cells. On both pure PCL scaffolds and PCL‐TCP composite scaffolds, metabolic activity increased between days 14 and 28, which suggests continued proliferation of the cells throughout the 4‐week culture period. Metabolic activity was higher in PCL‐TCP composite scaffolds than in pure PCL scaffolds at both time points.

### 3.2 PCL‐dECM scaffolds‐induced chondrogenic differentiation of hASC

**Figure 3:**
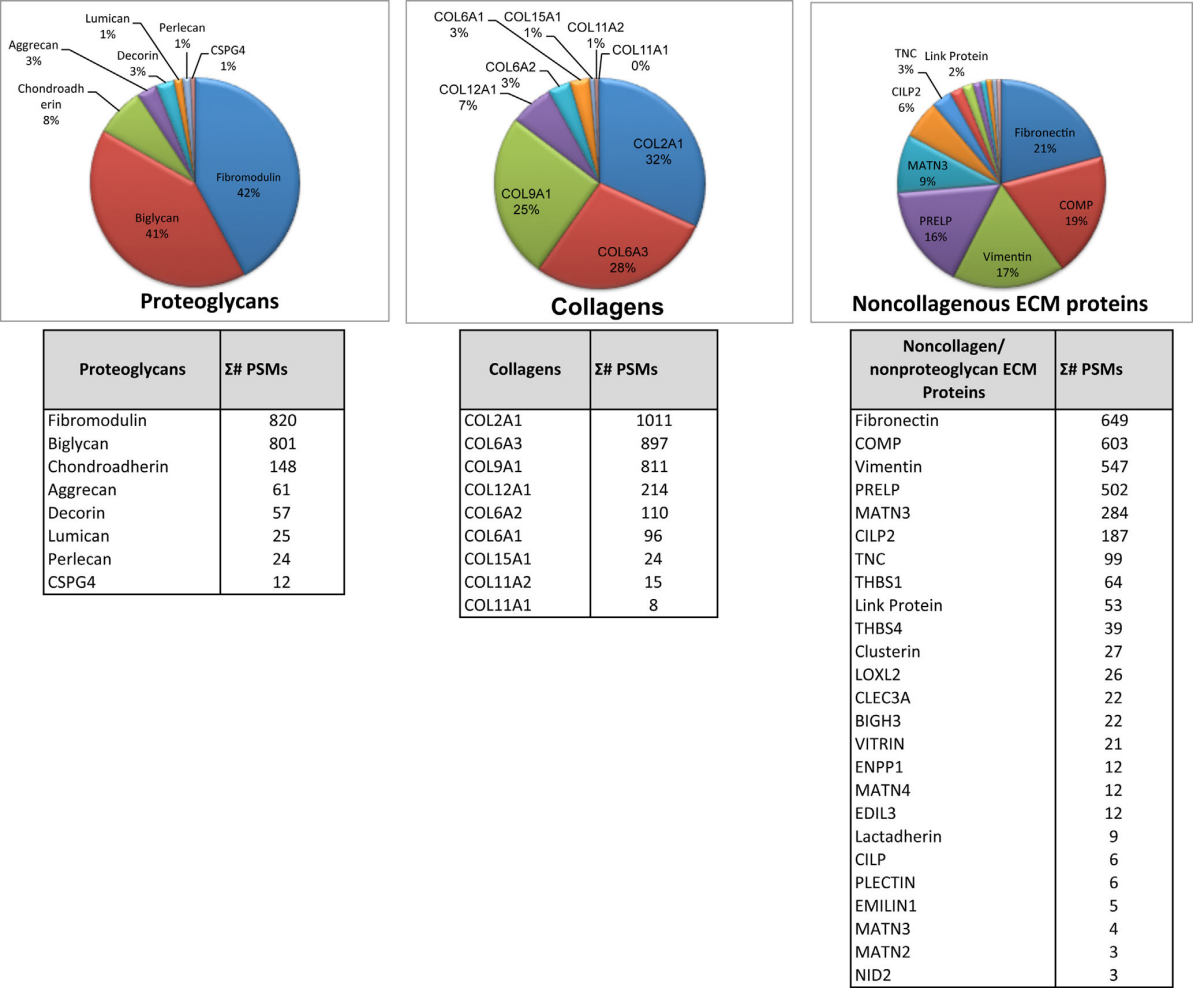
Proteomic analysis of decellularized bovine cartilage extracellular matrix (dECM). Proteomic analysis of the dECM identified several cartilage proteins based on 775 unique peptides from articular cartilage. Proteoglycans: The most prevalent include fibromodulin, biglycan, PRELP, chondroadherin, decorin, aggrecan, link protein, lumican, and perlecan. Collagens: The most prevalent identified include COL2A1, COL6A3, COL9A1, COL12A1, COL6A2, COL6A1, COL15A1, COL11A2, and COL11A1. Noncollagenous ECM proteins: The most prevalent include fibronectin, Cartilage Oligomeric Matrix Protein (COMP), matrilin 3 (MATN3), cartilage intermediate layer protein 2 (CILP2), tenascin C (TNC), thrombospondin type 1 (THBS1), thrombospondin type 4 (THBS4), and clusterin

The dECM hydrogel used to induce hASC chondrogenic differentiation was analyzed using proteomic analysis to ensure that the primary ECM proteins were preserved after the decellularization process (Pu & Oxford, 2015; Figure 3). We identified 775 unique peptides corresponding to proteoglycans, collagens, and other ECM proteins. Figure 3 shows the relative abundances of the most prevalent proteins.

**Figure 4:**
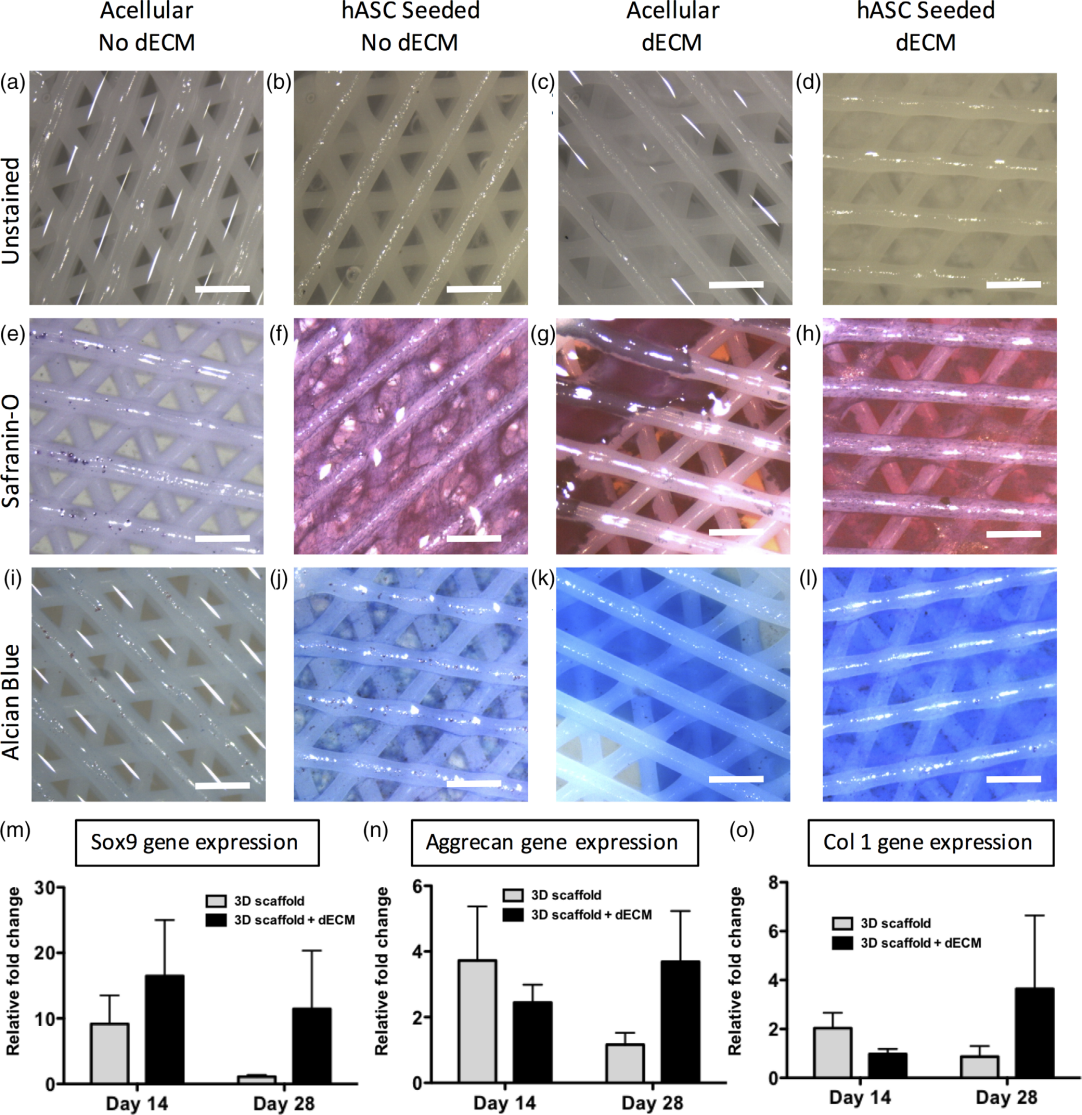
Chondrogenic evaluation of 3D‐bioplotted PCL‐dECM scaffolds. (a–d) Unstained scaffolds of each condition (e–h). Scaffolds stained for proteoglycan content via Safranin‐O. (i–l) Scaffolds stained for sulfated glycosaminoglycans content via Alcian Blue (scale bars = 200 μm). Gene expression of (m) sox9 and (n) aggrecan, and (o) collagen I. dECM, decellularized bovine cartilage extracellular matrix; PCL, polycraprolactone

Human ASC were then seeded on 3D‐bioplotted scaffolds with or without dECM and evaluated for chondrogenic differentiation. Histological staining showed more robust proteoglycan (Safranin‐O) and sGAG (Alcian Blue) staining in scaffolds that were seeded when compared to their unseeded controls, even in the absence of dECM (Figure 4). Unseeded scaffolds without dECM did not stain for either proteoglycan or sGAG content, as expected. Moderate matrix deposition was observed on the hASC seeded scaffolds without dECM, while the unseeded scaffolds with a dECM coating stained positive for proteoglycan or sGAG from the dECM itself. The unseeded dECM had regions of degradation and lacked organization. Seeded scaffolds with dECM coating had an evenly distributed matrix that had undergone remodeling from the cells. In addition, qualitative observation of the scaffolds showed that more matrix was deposited on the dECM seeded scaffolds than the unseeded scaffolds, indicating end product accretion of chondrogenic differentiation. Human ASC chondrogenesis was further evaluated by qPCR with similar results (Figure 4m–o). Although no statistical significance was observed in this data set, the following trends were observed. Seeded PCL‐dECM scaffolds exhibited increased expression of sox9, an early marker of chondrogenic differentiation (Kawakami, Rodriguez‐Leon, & Izpisua Belmonte, 2006). After 28 days in culture, aggrecan expression in PCL‐dECM scaffolds was elevated. Furthermore, Collagen I expression in dECM coated scaffolds was reduced relative to hASC seeded on non‐dECM scaffolds at day 14 but elevated at day 28.

### 3.3 Full thickness osteochondral scaffolds exhibited site‐specific tissue differentiation

**Figure 5:**
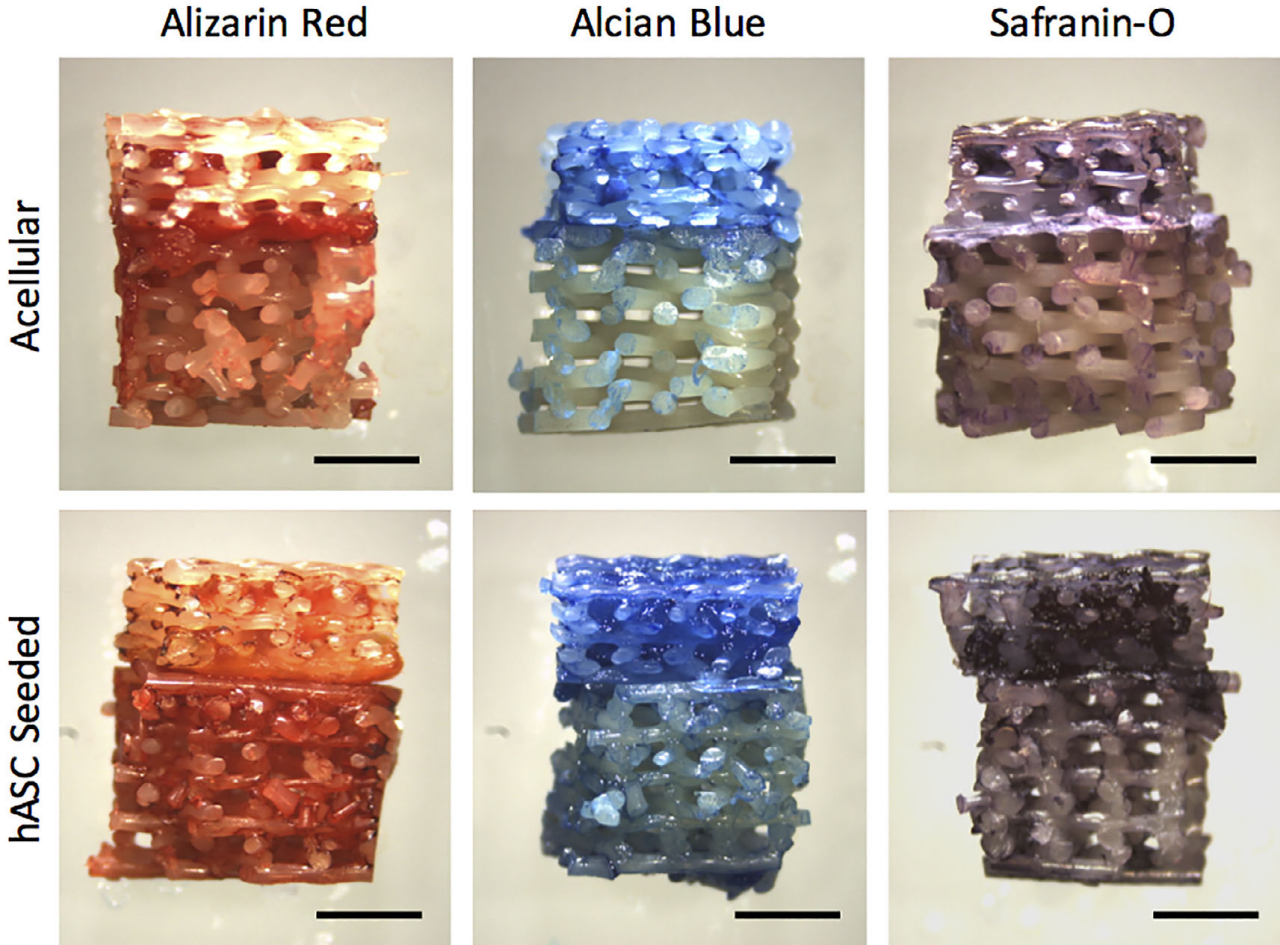
Site‐specific matrix distribution in full‐thickness osteochondral scaffolds. (a,b) Alizarin red shows calcium deposition in the osteo layer, while (c,d) Alcian Blue and (e,f) Safranin‐O staining demonstrate increased proteoglycan and sulfated glycosaminoglycans in the superficial cartilage layer (scale bars = 2 mm)

Histological staining of the full‐thickness scaffolds revealed site‐specific osteochondral tissue characteristics (Figure 5). The PCL‐TCP region of the scaffold stained dark red with the Alizarin Red staining, indicating calcium accretion within this region of the scaffold. The PCL‐dECM portion of the scaffold stained with Safranin‐O indicated the presence of proteoglycans, and Alcian Blue indicated the presence of sGAG. Alizarin Red, Alcian Blue, and Safranin‐O staining was more robust in the hASC seeded scaffolds than the nonseeded controls, demonstrating that end‐product expression rather than the biomimetic nature of the scaffolds themselves contributed to at least part of the observed staining patterns. In addition, the bone‐like portion of the scaffold and cartilage‐like portion of the scaffold transitioned cleanly from one phase to the other at the electrospun tide mark.

### 3.4 The electrospun tidemark prevents cell migration

**Figure 6:**
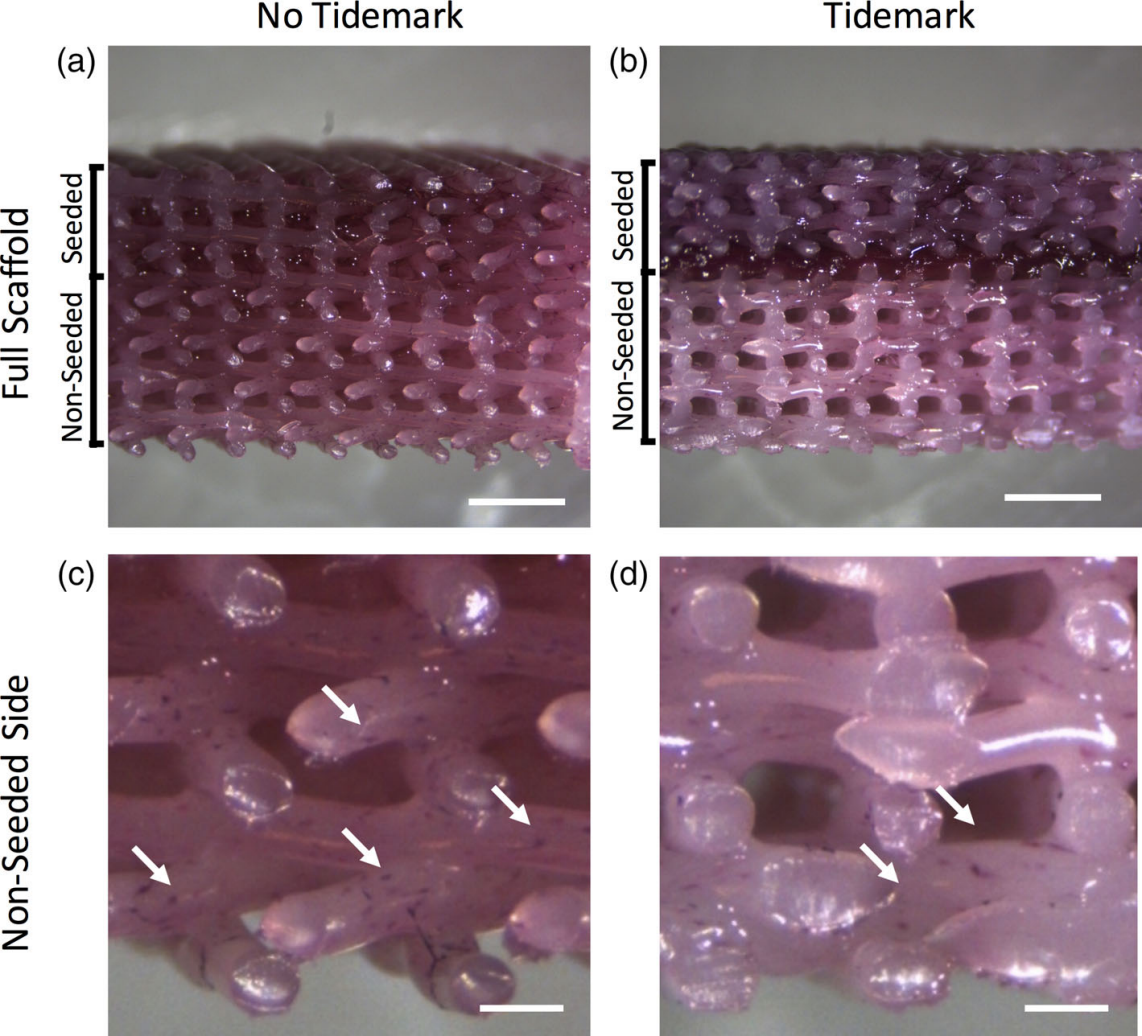
Gross comparison of scaffolds stained via hematoxylin and eosin shows that (a) scaffolds without an electrospun tidemark have relatively homogenous cellular distribution. (b) Scaffolds that contain an electrospun tidemark have more robust staining on the seeded side when compared to the nonseeded side of the scaffold. Higher magnification of the far nonseeded sides of the scaffolds demonstrates that more nuclei (arrows) are observed on the (c) scaffolds with no electrospun tidemark when compared to the (d) scaffolds containing an electrospun tidemark (scale bars = a,b: 2 mm, c,d: 400um)

H&E staining of scaffolds that were cultured with and without an electrospun layer to mimic a osteochondral tidemark displayed different cell distributions (Figure 6). Scaffolds that included a tidemark displayed much more robust nuclear and protein staining on the seeded side of the scaffolds and reduced cell nuclear and protein staining on the nonseeded side. However, scaffolds that did not include a tidemark had much more homogenous cell distribution throughout the scaffold. The scaffolds without an electrospun layer had a greater density of nuclei on the nonseeded side of the scaffold than the scaffolds that contained an electrospun layer.

## 4 DISCUSSION

The objective of this study was to develop a tissue engineered osteochondral construct that closely resembled the native architecture of osteochondral tissue using a single cell source of abundant and accessible hASC. A triphasic scaffold was created by combining two commonly used techniques (electrospinning and 3D printing), and incorporating chemical cues within the different layers of the scaffold to induce site‐specific osteogenic and chondrogenic differentiation of the hASC. Specifically, TCP was used to induce osteogenic differentiation and dECM was used to induce chondrogenic differentiation.

TCP has previously been shown to drive osteogenic differentiation (Asli, Pourdeyhimi, & Loboa, 2012; McCullen, Zhan, Onorato, Bernacki, & Loboa, 2010; Mellor et al., 2015). It has been demonstrated to induce differentiation in both 2D culture and 3D electrospun constructs (McCullen et al., 2010). More recently, we demonstrated that polylactic acid nanofibrous scaffolds containing 20% TCP nanoparticles drove osteogenic differentiation of hASC and that elevated calcium inhibited chondrogenic differentiation, even in the presence of chondrogenic growth factors (Mellor et al., 2015). Here, we confirm that scaffolds containing 20% TCP drive cells to differentiate down the osteogenic lineage by demonstrating that osteogenic markers are upregulated and end product calcium accretion is enhanced in the presence of TCP. When compared to previously reported electrospun scaffolds containing TCP, calcium is released at a much slower rate from the 3D‐bioplotted PCL‐TCP scaffolds. Specifically, we have previously demonstrated a burst release of 50% calcium within the first 24 hr in electrospun scaffolds (Asli et al., 2012). Here, we only witnessed a release of 0.54 μg (4.5% of total Ca^2+^) with the first 24 hr, and a sustained release over the course of 28 days. Even after 28 days, only 1.04 μg (8.7% of total Ca^2+^) had been released from the scaffolds, suggesting that calcium‐based signaling would be sustained long term in hASC seeded upon these scaffolds, which could be favorable for implantation of the tissue engineered constructs in vivo.

The cartilage layer of the 3D‐bioplotted scaffold was coated with cartilage dECM in order to drive chondrogenic differentiation of the hASC without the need of growth factor supplementation. In recent years, there has been great interest in the use of dECM for osteochondral tissue engineering (Benders et al., 2013). Since naturally derived ECM has a complex array of bioactive molecules, it is ideal to signal cells to adhere (Tavassoli, Matin, Niaki, Mahdavi‐Shahri, & Shahabipour, 2015), proliferate (Vorotnikova et al., 2010), and differentiate (Gong, Sagiv, Cai, Tsang, & Del Priore, 2008; Sellaro, Ravindra, Stolz, & Badylak, 2007). In cartilage tissue engineering, the use of a bioactive biomaterial is especially useful since cartilage is avascular and cannot easily circulate growth factors and nutrients (Benders et al., 2013). Previously, biphasic osteochondral scaffolds have been produced via combination of decellularized cartilage and decellularized bone (Yang et al., 2011). In addition, monophasic decellularized equine cartilage matrix implants have been used to generate biphasic osteochondral regeneration, although potential vascularization and ossification of the neo‐cartilage is a concern (Benders et al., 2013). In this study, we demonstrate that most of the crucial extracellular proteins of articular cartilage are preserved after the decellularization step. We used this naturally derived hydrogel to direct chondrogenic differentiation of hASC, even in the absence of supplemental chondrogenic growth factors. This is in accordance with previous literature, that demonstrated bone marrow‐derived mesencyhmal stem cells can be shunted down the chondrogenic lineage via stimulation with cartilage fragments without the use of growth factors (Chen et al., 2012). Additionally, in this study, we demonstrate that hASC have a synergistic effect with the dECM. We observe that seeded dECM displayed more robust proteoglycan and sGAG than the nonseeded dECM. Upon closer examination of the histology, the seeded dECM appeared to have been remodeled and augmented by the hASC whereas the nonseeded dECM suffered from degradation. This indicates that while the dECM is driving chondrogenic differentiation, the cells are simultaneously modifying the structural properties of the dECM. The results from both dECM and TCP studies drove the development of single osteochondral scaffolds, which can be cultured in CGM alone (without any additional osteogenic and/or chondrogenic growth factors) to induce both hASC chondrogenesis and osteogenesis in desired site‐specific locations.

A novel aspect of our triphasic design was the incorporation of a thin eletropsun PCL layer between the osteogenic and chondrogenic layers to mimic the natural tidemark found in osteochondral tissue. When engineering osteochondral implants, it is a concern that the neo‐cartilage would ossify if implanted in vivo long term (Benders et al., 2013). The incorporation of the electrospun tidemark layer is important because it prevents cell migration between the cartilage and bone layers. The in vitro results of this study indicate that hASC migration is inhibited in scaffolds that include an electrospun layer. This layer could potentially act as a barrier to prevent blood vessel invasion into the cartilage layer when implanted in vivo.

Since the overall objective of this study was to generate an osteochondral implant with site‐specific characteristics, the global protein and calcium distributions within the 3D multiphasic scaffolds were assessed. The scaffolds were cultured for 4 weeks, which gave the bioactive factors and cells seeded upon those layers ample time to interact with each other. Since both the osteogenic side and chondrogenic side were cultured simultaneously within the same dish, interactions between the two phases cannot be avoided, and we did not attempt to mitigate these interactions. Since bone and cartilage are known to crosstalk extensively in vivo (Funck‐Brentano & Cohen‐Solal, 2011), this mimics native osteochondral signaling. Staining revealed that site‐specific characteristics within the 3D architecture are maintained throughout the duration of the culture, despite these interactions.

Although the current study presents a novel system for producing site‐specific differentiation of hASC within a single scaffold, a limitation of the current study is that only in vitro characterization was performed. Although in vitro characterization is an essential step before moving into animal models, this scaffold system will require in vivo validation in future studies. Specifically, in order to assess the potential for large scale preclinical studies and eventual clinical translation, this scaffold system must be validated in a large animal model to ensure that the scaffold does not elicit an immune response and achieves the desired healing in vivo (Cook et al., 2014). Additionally, for this study we use decellularized porcine ECM as it is easily obtainable and is amenable to future porcine in vivo studies. However, for clinical purposes, large amounts of human cartilage will be more difficult to obtain, and therefore an immune response to porcine tissue will have to be considered. Although the tissue is decellularized and sterilized, further studies to assess cross‐species immune response will be necessary prior to clinical studies.

This study is the first to incorporate TCP and dECM in a single 3D‐bioplotted scaffold to induce site‐specific differentiation of hASC as a single cell source to generate a full osteochondral construct. Recent research in osteochondral scaffold fabrication has utilized 3D printing approaches to generate patient‐specific scaffolds in a precise and highly tunable manner. In this study, we take advantage of recent advances in biofabrication to generate scaffolds that are thick enough to repair full‐thickness osteochondral defects, unlike electrospun scaffolds. We show, for the first time, that the incorporation of an electrospun layer can inhibit cell migration between scaffold layers, which may be highly advantageous in vivo by preventing blood vessels from invading the chondrogenic portion of the scaffold. The scaffold design described in this study minimizes rejection by using an abundant and accessible source of autologous stem cells, and our biofabrication techniques allow for a precise, customizable methodology to rapidly produce the scaffold. With further development, this novel approach could hold great potential to treat OA patients by offering a patient‐specific, less‐invasive alternative to full joint replacement.
